# Mycobacterial Antigen Driven Activation of CD14^++^CD16^−^ Monocytes Is a Predictor of Tuberculosis-Associated Immune Reconstitution Inflammatory Syndrome

**DOI:** 10.1371/journal.ppat.1004433

**Published:** 2014-10-02

**Authors:** Bruno B. Andrade, Amrit Singh, Gopalan Narendran, Melissa E. Schechter, Kaustuv Nayak, Sudha Subramanian, Selvaraj Anbalagan, Stig M. R. Jensen, Brian O. Porter, Lis R. Antonelli, Katalin A. Wilkinson, Robert J. Wilkinson, Graeme Meintjes, Helen van der Plas, Dean Follmann, Daniel L. Barber, Soumya Swaminathan, Alan Sher, Irini Sereti

**Affiliations:** 1 Immunobiology Section, Laboratory of Parasitic Diseases, National Institute of Allergy and Infectious Diseases, National Institutes of Health, Bethesda, Maryland, United States of America; 2 Clinical and Molecular Retrovirology Section, Laboratory of Immunoregulation, National Institute of Allergy and Infectious Diseases, National Institutes of Health, Bethesda, Maryland, United States of America; 3 National Institute for Research in Tuberculosis, Chennai, India; 4 Laboratório de Imunopatologia, Centro de Pesquisas René Rachou, Fundação Oswaldo Cruz, Belo Horizonte, Minas Gerais, Brazil; 5 Clinical Infectious Diseases Research Initiative, Institute of Infectious Disease and Molecular Medicine, and Department of Medicine, University of Cape Town, Cape Town, South Africa; 6 Department of Medicine, Imperial College London, London, United Kingdom; 7 MRC National Institute for Medical Research, London, United Kingdom; 8 Biostatistics Research Branch, National Institute of Allergy and Infectious Diseases, National Institutes of Health, Bethesda, Maryland, United States of America; 9 T-Lymphocyte Biology Unit, Laboratory of Parasitic Diseases, National Institute of Allergy and Infectious Diseases, National Institutes of Health, Bethesda, Maryland, United States of America; Emory University, United States of America

## Abstract

Paradoxical tuberculosis-associated immune reconstitution inflammatory syndrome (TB-IRIS) is an aberrant inflammatory response occurring in a subset of TB-HIV co-infected patients initiating anti-retroviral therapy (ART). Here, we examined monocyte activation by prospectively quantitating pro-inflammatory plasma markers and monocyte subsets in TB-HIV co-infected patients from a South Indian cohort at baseline and following ART initiation at the time of IRIS, or at equivalent time points in non-IRIS controls. Pro-inflammatory biomarkers of innate and myeloid cell activation were increased in plasma of IRIS patients pre-ART and at the time of IRIS; this association was confirmed in a second cohort in South Africa. Increased expression of these markers correlated with elevated antigen load as measured by higher sputum culture grade and shorter duration of anti-TB therapy. Phenotypic analysis revealed the frequency of CD14^++^CD16^−^ monocytes was an independent predictor of TB-IRIS, and was closely associated with plasma levels of CRP, TNF, IL-6 and tissue factor during IRIS. In addition, production of inflammatory cytokines by monocytes was higher in IRIS patients compared to controls pre-ART. These data point to a major role of mycobacterial antigen load and myeloid cell hyperactivation in the pathogenesis of TB-IRIS, and implicate monocytes and monocyte-derived cytokines as potential targets for TB-IRIS prevention or treatment.

## Author Summary

Tuberculosis and HIV majorly impact host immune responses, resulting in immune deregulation and inflammation-driven tissue damage. Initiation of anti-retroviral therapy in patients with HIV-TB co-infection may result in immune reconstitution inflammatory syndrome (TB-IRIS), a disorder associated with increased immunopathology due to unfettered inflammation after CD4^+^ T-cell reconstitution. Monocytes are critical to the innate immune system and play an important role in several inflammatory conditions associated with chronic infections. Immunopathogenesis of TB-IRIS has been linked to activation of the adaptive immune response against opportunistic infection, yet the role of monocytes is still unknown. Here we investigated associations between soluble markers of monocyte activation, differential activation of monocyte subsets and TB-IRIS prospectively in two geographically distinct HIV-TB co-infected patient cohorts. Prior to ART initiation, patients who developed IRIS displayed a biosignature of elevated soluble monocyte activation markers, which were closely related to the mycobacterial antigen load in sputum samples. Amongst monocyte subsets, we observed that pre-ART circulating CD14^++^CD16^−^ cell frequency independently predicted TB-IRIS and expanded during IRIS events. This monocyte subset was tightly associated with systemic markers of inflammation, and was found to produce inflammatory cytokines. Identification of this monocyte subset and its link with inflammation may lead to conception of novel therapies reducing immunopathology in TB-IRIS.

## Introduction

Implementation of antiretroviral therapy (ART) in patients co-infected with HIV and tuberculosis (TB) has greatly improved life expectancy [1]–[5]. Anti-retroviral therapy reconstitutes the number and function of CD4^+^ T-cells and most patients manifest clinical improvement of signs and symptoms of opportunistic co-infections including tuberculosis (TB). Nevertheless, some patients experience a paradoxical worsening of TB during the first 3 months of ART, a phenomenon known as immune reconstitution inflammatory syndrome or IRIS [6]–[8]. The incidence of TB-IRIS is variable (from 8 to 54%) depending on the epidemiological settings [9]–[14]. The clinical manifestations can range from fever and lymph node enlargement to sepsis-like syndrome and neurological deterioration [7]. The immunological basis of the pathological mechanisms leading to TB-IRIS is still not fully understood. The clinical onset of IRIS has been linked to hyperactivation of T-cells specific for antigens from opportunistic pathogens, resulting in an inflammatory cytokine storm [15]–[20]. In addition, different components of the innate immune response such as natural killer cells (NK) [21], macrophages [22], monocytes [23], [24] and neutrophils [25] have been implicated in the pathogenesis of TB-IRIS [26]. Monocytes are critical for host immunity against both TB and HIV, and as such, their specific role in TB-IRIS warrants better understanding. In a recent study of TB-IRIS, transcriptional analysis of monocytes suggested a potential role of monocytes and the complement system in this phenomenon [24]. In addition, myeloid cells and innate cytokines such as IL-6 have been proposed to play a role in mycobacterial IRIS, as suggested in an animal model of IRIS developed by our group [27], [28].

Human monocyte subpopulations can be categorized based on the dichotomous expression of the surface markers CD14 and CD16 into three major subsets: CD14^++^CD16^−^, CD14^+^CD16^+^ and CD14^dim^CD16^+^. These different monocyte subsets have been described to exhibit very distinct functional roles in a range of homeostatic and pathological conditions [29]. No detailed analysis of the different monocyte subsets in TB-IRIS has been performed in patients. The goal of the present study was to evaluate the role of monocyte subsets and their association with pro-inflammatory cytokines in the setting of TB-IRIS. Our findings from studying three geographically distinct patient cohorts demonstrate that expansion and activation of the CD14^++^CD16^−^ monocyte subset is closely associated with the pathogenesis of TB-IRIS.

## Results

### Paradoxical TB-IRIS is characterized by high plasma levels of inflammatory biomarkers related to monocytes

**Figure 1:**
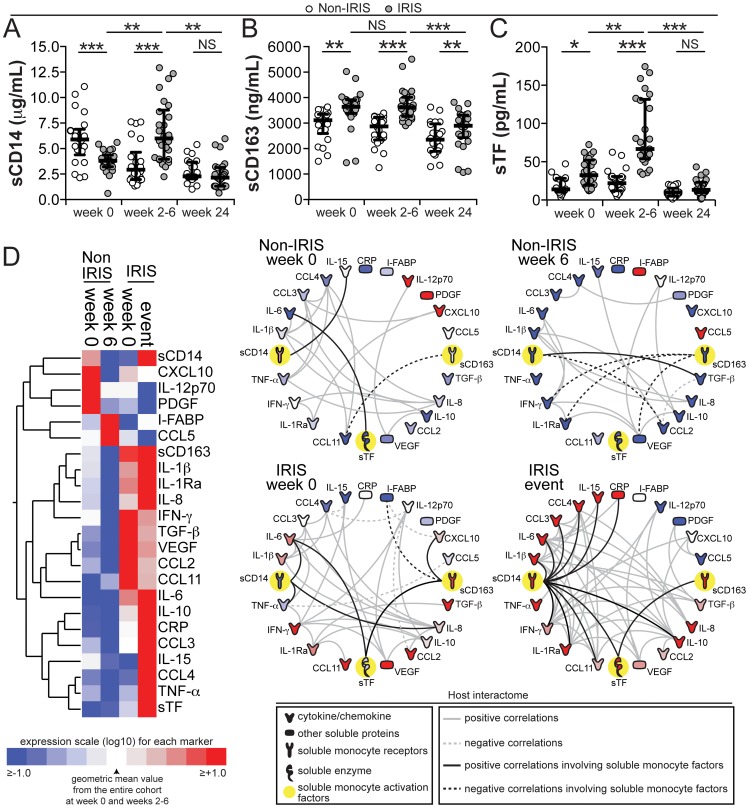
Plasma concentrations of monocyte activation markers are increased during TB-IRIS and correlate with markers of systemic inflammation. (**A–C**) Plasma levels of soluble (s) CD14 (**A**), sCD163 (**B**) and sTF (**C**) are compared at week 0 (pre-ART), at week 6 or at the time of IRIS, and at week 24 after ART initiation between TB-HIV co-infected patients who developed paradoxical TB-IRIS (n = 26) and those who did not (n = 22). Lines represent median values and interquartile ranges. Data were analyzed using Mann-Whitney or Wilcoxon matched-pairs test for paired analyses within each study group. * P<0.05, ** P<0.01, *** P<0.001. (**D**) Left panel: A heat map was designed to depict the overall pattern of expression of plasma cytokines, chemokines and inflammatory markers in patients developing TB-IRIS vs. non-IRIS controls at week 0 (pre-ART) and at week 6 or during the time of IRIS. A two-way hierarchical cluster analysis (Ward's method) of circulating biomarkers by clinical group and time point was performed. Expression scale for each biomarker represents log_10_ fold-change from the geometric mean of the entire study population at week 0 and week 6 or time of IRIS (n = 48 in each study time point). Right panel: The network analysis (interactome) shows statistically significant correlations (P<0.05) between all the variables measured. Data were analyzed using Spearman rank tests. See Data File S1 for additional details on the strength (r value) and level of significance (P-value) of each individual correlation.

TB-IRIS has been clearly shown to be associated with elevated plasma concentrations of several pro-inflammatory and anti-inflammatory cytokines [20], some of them known to be strongly linked to monocyte stimulation. We tested whether the monocyte and macrophage activation markers sCD14, sCD163 and sTF were also altered in TB-IRIS in a cohort of patients from South India. We found significantly higher levels in TB-IRIS patients vs. non-IRIS controls at baseline of sCD163 (median 3638 ng/mL, IQR: 3411–3880 ng/mL vs. 3257 ng/mL, IQR: 2823–3343 ng/mL, P<0.001) and sTF (median 32.5 pg/mL, IQR: 19.3–52.1 pg/mL vs. 13.9 pg/mL, IQR: 10.2–28.2 pg/mL, P<0.001), whereas sCD14 was significantly lower at baseline (median 3.8 µg/mL, IQR: 3.2–4.4 µg/mL vs. 5.5 µg/mL, IQR: 4.4–6.7 µg/mL, P<0.001). At the time of IRIS, levels of all three markers were higher in IRIS patients compared to non-IRIS controls (Figure 1). In addition, statistically significant differences were observed when comparing changes from baseline to the time of IRIS or an equivalent time point in the IRIS vs. non-IRIS groups for sCD14 (median fold-change from baseline 1.2, IQR: 0.64–1.7 vs. 0.6, IQR: 0.45–0.9, P = 0.012), sCD163 (median fold-change from baseline 1.0, IQR: 0.94–1.1 vs. 0.94, IQR: 0.85–1.0, P = 0.015) and sTF (median fold-change from baseline 2.4, IQR: 2.1–2.9 vs. 1.07, IQR: 0.76–2.5, P<0.001). At 24 weeks after ART initiation, levels of these biomarkers significantly decreased in both IRIS and non-IRIS patients, with no significant differences between the groups except for sCD163 levels, which remained higher in the IRIS group (Figure 1A–C).

We next compared plasma levels of several pro- and anti-inflammatory cytokines, chemokines and markers of tissue damage in the Indian cohort. Consistent with previous reports [20], we confirmed that after ART initiation, patients who experienced IRIS exhibited elevated levels of many pro- and anti-inflammatory biomarkers with 7 out of the 16 cytokines and chemokines measured dramatically elevated in TB-IRIS patients (Figure 1D and Table S1). Nevertheless, circulating levels of CXCL10, IL-12p70 and PDGF were higher in non-IRIS control patients (Figure 1D and Table S1). In addition, after 6 weeks of ART, individuals without IRIS events had higher plasma concentrations of I-FABP and CCL5 (Figure 1D and Table S1).

**Figure 2:**
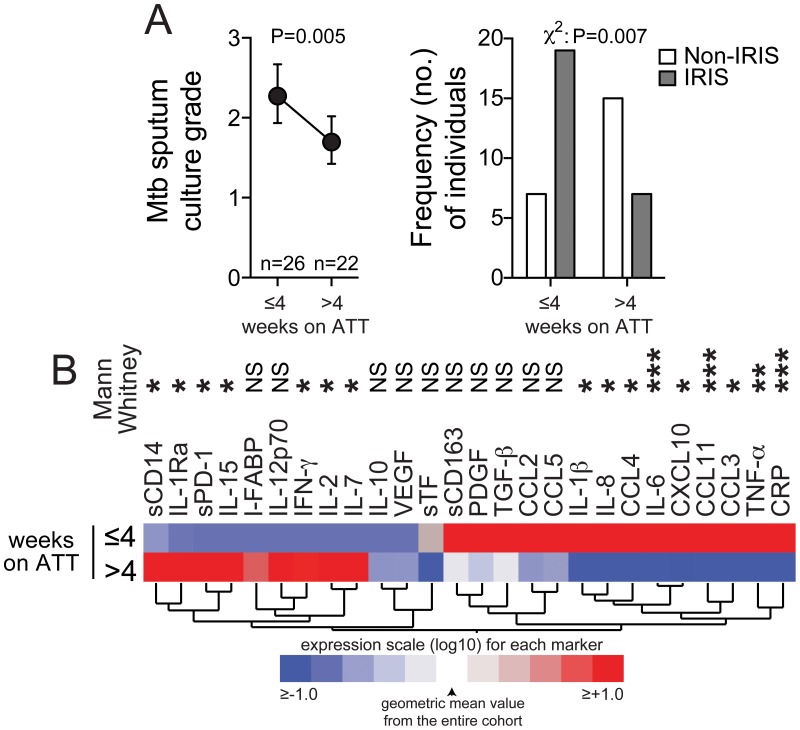
Expression profile of plasma biomarkers of inflammation and immune activation in TB-HIV co-infected patients on anti-TB therapy. (**A**) Left panel shows *Mycobacterium tuberculosis* sputum culture grade in samples from TB-HIV co-infected individuals at study enrollment (pre-ART) stratified by time on anti-TB treatment (ATT). Data were compared using the Mann-Whitney test. Right panel shows the frequency of IRIS vs. non-IRIS patients taking ATT for different durations (≤4 weeks and>4 weeks). Data were compared using the Chi-square test. (**B**) A heat map was designed to depict the overall pattern of expression of plasma cytokines, chemokines and inflammatory biomarkers in TB-HIV co-infected patients at different time points after ATT initiation. A hierarchical cluster analysis (Ward's method) of circulating biomarkers by clinical group and time point was performed. Expression scale for each biomarker represents change from the geometric mean of the entire study population (n = 48). Differences between medians were compared using the Mann-Whitney test. * P<0.05, ** P<0.01, *** P<0.001.

As reported in other studies, a critical difference between the patients who developed IRIS and those who did not was duration of anti-TB therapy (ATT) before ART initiation at study baseline: median of 20 days (IQR: 14–30 days) in TB-IRIS cases versus 43 days (IQR: 23–68 days) in controls (P = 0.002) [30]. In order to assess how the duration of ATT impacts the cytokine and chemokine profile of patients prior to ART (a factor that has been repeatedly associated with risk of IRIS), we compared the concentrations of plasma biomarkers in study patients grouped according to time on ATT (Figure 2). As expected, the mycobacterial load in sputum cultures significantly reduced with time on ATT (P = 0.005; Figure 2A). The pattern of distribution of plasma biomarkers also changed significantly with duration of ATT. Individuals who were on ATT for less than 4 weeks exhibited higher concentrations of plasma biomarkers associated with activation of innate immune responses, such as IL-1β, IL-6, CRP, sCD163, IL-8, CCL2, PDGF as well as several chemokines (Figure 2B). In contrast, biomarkers that are more closely associated with activation of adaptive immune responses, such as IFN-γ, IL-2, IL-15, sPD-1 and IL-7, were higher in patients who were on ATT for more than 4 weeks before ART initiation. The finding that IRIS patients received ATT for a shorter time before ART initiation (Figure 2A) suggested the importance of an elevated Mtb antigen load, as supported by increased colony forming unit grades in sputum Mtb cultures.

### The plasma inflammatory profile of TB-IRIS patients is not associated with absolute counts of monocytes or neutrophils in the peripheral blood

To further investigate the association between monocyte activation markers and systemic inflammation in TB-IRIS, we employed a network analysis based on multiple Spearman correlations between all the biomarkers measured in plasma (Figure 1D and Data File S1). We found that in IRIS patients, the number of significant correlations between multiple variables was very high compared to non-IRIS controls (P<0.001; Figure 1D, Data File S1 and Figure S2). This finding from network analysis argues that the cytokine storm observed in TB-IRIS is tightly coordinated. In addition, the direction of change in network densities observed in the IRIS vs. non-IRIS groups did not seem to be associated with HIV viral load or CD4^+^ T-cell count (Figure S2), implying that other factors may affect more directly how plasma biomarkers correlate with one another.

**Figure 3:**
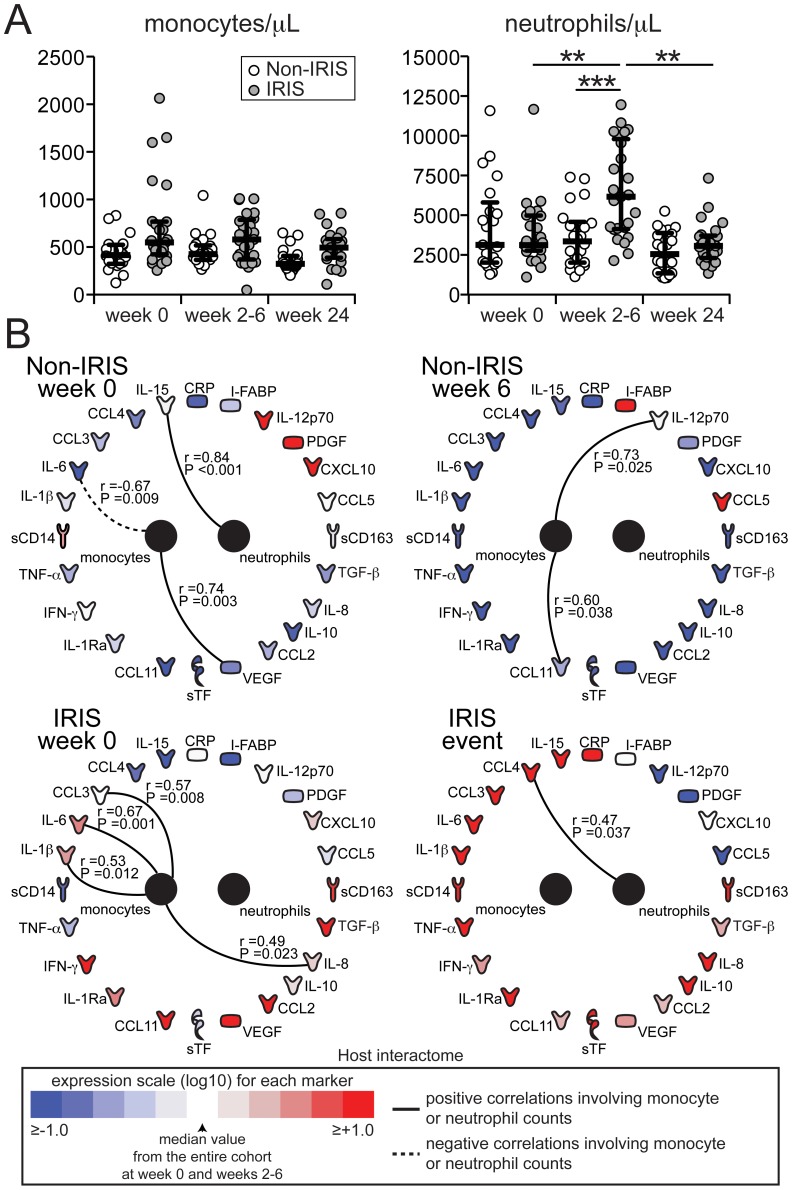
Circulating monocyte counts do not correlate with soluble biomarkers of inflammation and immune activation in TB-HIV co-infected individuals. (**A**) Numbers of circulating monocytes (left panel) and neutrophils (right panel) are compared at week 0 (pre-ART), at weeks 6 or at the time of IRIS, and at week 24 after ART initiation between TB-HIV co-infected patients who developed paradoxical TB-IRIS (n = 26) and those who did not (n = 22). Lines represent median values and interquartile ranges. Data were analyzed using the Mann-Whitney test or Wilcoxon matched-pairs test for paired comparisons within each study group. ** P<0.01, *** P<0.001. (**B**) The network analysis (interactome) showed statistically significant correlations (P<0.05) between neutrophil or monocyte counts and plasma biomarkers. Associations were assessed with Spearman rank tests.

Our results from measurements of multiple plasma biomarkers suggest that biomarkers of monocyte activation are associated with the inflammatory environment of TB-IRIS after ART initiation. We next tested whether the concentration of monocytes in peripheral blood reflected the observations from plasma protein analysis and found no significant differences in total monocyte counts between the study groups at the different time points evaluated (Figure 3A). Absolute neutrophil counts were higher in the IRIS group at week 6 compared to non-IRIS controls (P<0.001; Figure 3A). However, the network analysis revealed a lack of significant correlations between absolute neutrophil and monocyte counts and the plasma biomarkers of inflammation at all time points studied (Figure 3B). At enrollment (pre-ART), patients who developed IRIS during the study displayed significant positive correlations between monocyte counts and innate cytokines such as IL-1β (r = 0.53, P = 0.012), IL-6 (r = 0.67, P = 0.001) and IL-8 (r = 0.49, P = 0.023) (Figure 3B).

**Figure 4:**
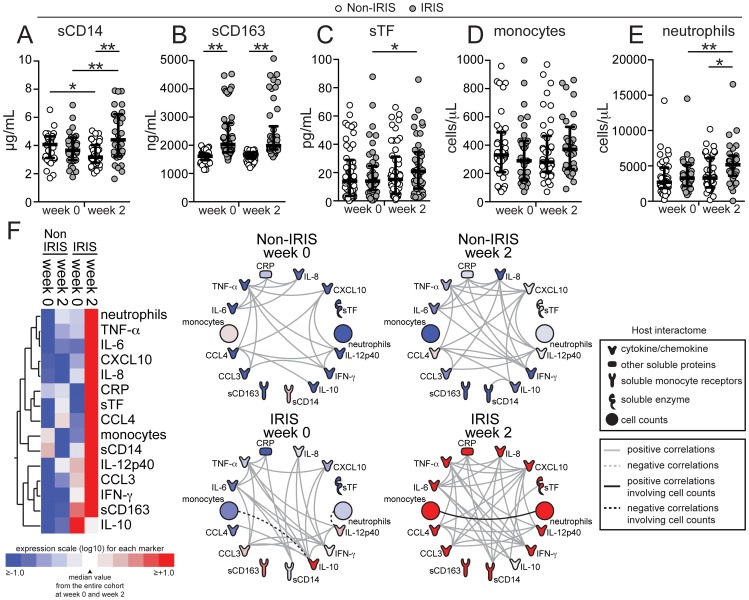
TB-HIV co-infected patients from South Africa exhibited a similar inflammatory biomarker pattern of paradoxical TB-IRIS as well as a lack of correlation between monocyte counts and soluble biomarkers of inflammation. (**A–D**) Plasma levels of sCD14 (**A**), sCD163 (**B**), sTF (**C**), absolute monocyte count (**D**), and absolute neutrophil counts (**E**) were compared at week 0 (pre-ART) and at week 2 after ART initiation between TB-HIV co-infected patients that developed paradoxical TB-IRIS (n = 44) and those who did not (n = 52). Lines represent median values and interquartile ranges. Data were analyzed using the Mann-Whitney test or Wilcoxon matched-pairs test for paired comparisons within each study group. * P<0.05, ** P<0.01. (**F**) Left panel: A Heat map was designed to depict the overall pattern of expression of plasma cytokines, chemokines and inflammatory biomarkers in IRIS vs. non-IRIS patients at week 0 (pre-ART) and at week 2. A two-way hierarchical cluster analysis (Ward's method) of circulating biomarkers by clinical group and time point was performed. Expression scale for each biomarker represents log_10_ fold-change from the geometric mean of the entire study population at week 0 and week 2 or the time of IRIS (n = 96 at each study time point). Right panel: The network analysis (interactome) shows statistically significant correlations (P<0.05) between all the parameters measured. Data were analyzed using Spearman rank tests. See Data File S2 for additional details on the strength (r value) and level of significance (P-value) of each individual correlation.

To test whether the findings from our Indian cohort could be validated in another area endemic for TB-HIV co-infection, we performed similar analyses of plasma biomarkers and absolute cell counts in a cohort of HIV-TB co-infected patients from South Africa, which has been previously described [31], [32]. Levels of sCD14 were not significantly different between the groups at baseline (pre-ART) (P = 0.502) and similarly to what was observed in the South Indian cohort, the values were significantly higher at week 2 in the IRIS group (P = 0.003; Figure 4A and Table S2). Levels of sCD163 were again higher before ART initiation in patients who developed IRIS compared to those who did not (median 1947 ng/mL, IQR: 1543–2582 ng/mL vs. 1612 ng/mL, IQR: 1473–1692 ng/mL, P = 0.001) and remained elevated at the time of IRIS without a significant change from baseline (Figure 4B). Pre-ART plasma levels of sTF did not differ (14.5 pg/mL, IQR: 7.7–24.7 pg/mL vs. 13.9 pg/mL, IQR: 3.6–28.9 pg/mL, P = 0.078), but were increased upon ART initiation in IRIS patients, whereas they did not change in non-IRIS patients (median fold-change from baseline 1.25, IQR: 0.89–1.63 vs. 0.69, IQR: 0.47–1.02, P = 0.036, Figure 4B). Monocyte counts remained unchanged upon ART initiation in both groups (Figure 4C), whereas absolute neutrophil counts were higher during the IRIS event in the IRIS group (median 5161 cells/µL, IQR: 3590–6460 cells/µL vs. 3375 cells/µL, IQR: 2065–6595 cells/µL, P = 0.018, Figure 4D). In addition, further analysis using measurements of all the biomarkers available in the South African cohort validated the following findings from the Indian cohort: (i) plasma concentrations of most of the inflammatory biomarkers were increased during the time of IRIS (Figure 4, Table S2); (ii) the number of statistically significant correlations was greatly increased during the time of IRIS compared to matched time points in non-IRIS patients, as revealed by network analysis (P<0.001; Figure 4E, Figure S2 and Data File S2); and (iii) there was a lack of significant correlations between absolute neutrophil and monocyte counts and plasma biomarkers (Figure 4E).

### Expansion of circulating CD14^++^CD16^−^ monocytes with high expression of CD163 and lack of CD14^dim^CD16^+^ cells in TB-IRIS

**Figure 5:**
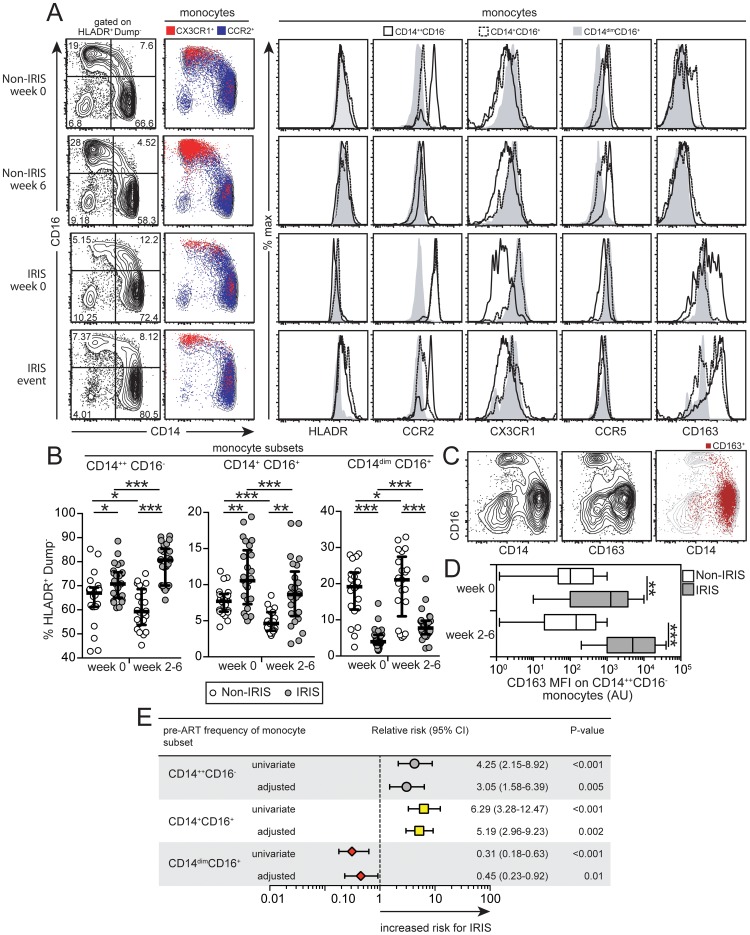
Dynamics of circulating monocyte subsets in TB-HIV co-infected patients following ART initiation. (**A**) Left panel: Representative FACS plots showing the distribution of the monocyte subsets defined by CD14 and CD16 markers at week 0 and at week 6 or time of IRIS in IRIS vs. non-IRIS patients. Monocytes were also defined according to expression of CCR2 and CX3CR1 and overlaid onto CD14 vs. CD16 graphs. Right panel: Representative FACS plots showing histograms of various phenotypic markers in monocyte subsets based on CD14 and CD16 expression by clinical group and time point. (**B**) Percentage of circulating CD14^++^CD16^−^ (left), CD14^+^CD16^+^ (center) and CD14^dim^CD16^+^ (right) monocytes among circulating mononuclear myeloid cells (HLADR^+^CD2^−^CD3^−^CD19^−^CD20^−^CD56^−^) were compared at week 0 (pre-ART) and at week 6 or at the time of IRIS after ART initiation between TB-HIV co-infected patients that developed paradoxical TB-IRIS (n = 26) and those who did not (n = 22). Lines represent median values and interquartile ranges. Data were analyzed using the Mann-Whitney test or Wilcoxon matched-pairs test for paired analyses within each study group. (**C**) Representative FACS plots showing co-localization of CD163 and CD14 in circulating monocytes from an IRIS patient at week 2 of ART. (**D**) The median fluorescence intensity (MFI) of CD163 expression on CD14^++^CD16^−^ monocytes is compared at week 0 (pre-ART) and week 6 or at the time of IRIS after ART initiation between TB-HIV co-infected patients that developed paradoxical TB-IRIS (n = 26) and those who did not (n = 22) using the Mann-Whitney test. (**E**) Relative risk (RR) of developing paradoxical TB-IRIS per standard deviation increase in the frequency of specific monocyte subsets at week 0 (pre-ART) after log_10_ transformation. RR were adjusted for baseline age, gender, days to ART initiation, plasma HIV RNA levels and CD4^+^ T-cell count. CI, confidence interval. * P<0.05, ** P<0.01, *** P<0.001.

Our results from two separate areas endemic for HIV-TB co-infection suggest that soluble markers of monocyte/myeloid activation are associated with the occurrence of paradoxical TB-IRIS following ART initiation, but total monocyte or neutrophil counts do not seem to influence this association. We then hypothesized, that differences in monocyte subsets rather than total monocyte counts could explain the results from plasma analyses. To address this, we performed multicolor flow cytometry experiments in whole blood for phenotypic analysis of monocyte subsets from patients in the Indian cohort (Figure S1). At study baseline and also after ART initiation, individuals who developed IRIS exhibited higher frequencies vs. non-IRIS patients of CD14^++^CD16^−^ (median 73.2% of circulating mononuclear myeloid cells, IQR: 64.7–75.1% vs. 65.6%, IQR: 60.9–68.8%, P = 0.029) and CD14^+^CD16^+^ (median 10.4% of circulating mononuclear myeloid cells, IQR: 7.2–14.5% vs. 7.6%, IQR: 6.2–8.6%, P = 0.008) monocyte subsets, whereas the CD14^dim^CD16^+^ monocyte subset was significantly decreased (median 3.9% of circulating mononuclear myeloid cells, IQR: 3.1–5.8% vs. 18.9%, IQR: 12.6–22.7%, P<0.001) (Figure 5A–B).

Analyses of the monocyte subsets confirmed that the chemokine receptor CX3CR1 was more highly expressed in the CD14^dim^CD16^+^ subset, whereas CCR2 was more prevalent in the CD14^++^CD16^−^ and CD14^+^CD16^+^ subsets (Figure 5A). The CD14^dim^CD16^+^ subset also displayed slightly reduced (albeit not significantly different) expression of HLA-DR and CCR5 compared with other monocyte subsets at the time of IRIS event, as assessed by mean fluorescence intensity (MFI) (Figure 5A). Among the surface markers evaluated, CD163 displayed marked variation in expression, especially in the CD14^++^CD16^−^ subset (Figure 5A–D). CD163 expression co-localized with CD14 on the surface of monocytes (Figure 5C) and was elevated pre-ART on CD14^++^CD16^−^ monocytes in IRIS patients compared with non-IRIS controls (MFI 1271 arbitrary units [AU], IQR: 100.1–3696 AU vs. 101.5 AU, IQR: 47.5–425.0 AU, P = 0.002; Figure 5D), being further increased at the time of IRIS (MFI 5000.3 AU, IQR: 1000.5–20050.6 AU at IRIS event vs. 146.0 AU, IQR: 20.3–492.5 AU at week 6 in non-IRIS controls, P<0.001; Figure 5D). Moreover, CD163 expression on the CD14^++^CD16^−^ subset was positively correlated with plasma levels of sCD163 (Spearman r = 0.63, P<0.001), suggesting that this monocyte subset could be a source of the soluble receptor.

We next analyzed the changes in each of the major monocyte and other mononuclear myeloid subsets between study baseline and the time of IRIS or 6 weeks post-ART initiation in non-IRIS patients. The total number of circulating mononuclear myeloid cells did not significantly change in IRIS (34.7±12.5% of total PBMC) or non-IRIS patients (35.2±9.3% of total PBMC) (Figure S3A). Among all monocytes, the CD14^++^CD16^−^ subset exhibited the greatest increase in IRIS patients and the greatest decrease in non-IRIS patients after ART initiation (median delta variation for IRIS vs. non-IRIS groups: 9.9%, IQR: 8.5 to 11.5% vs. −7.7%, IQR: −5.7 to −8.2%, P<0.001; Figure S3B). Multivariate logistic regression analysis adjusted for baseline age, gender, days to ART initiation, plasma HIV RNA levels and CD4^+^ T-cell count revealed that the frequency of each circulating monocyte subset prior to ART initiation was an independent predictor of TB-IRIS developing during the study (Figure 5E).

**Figure 6:**
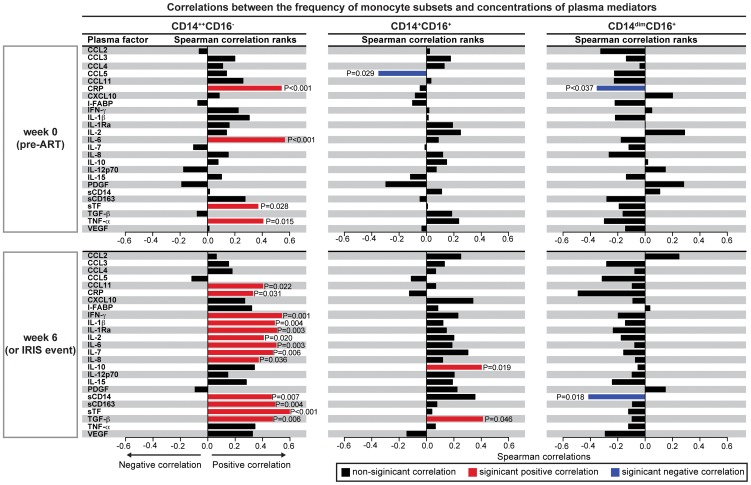
The CD14^++^CD16^−^ monocyte subset strongly correlates with pro-inflammatory biomarkers in TB-HIV co-infected individuals upon ART initiation. Percentage of circulating CD14^++^CD16^−^ (left), CD14^+^CD16^+^ (center) and CD14^dim^CD16^+^ (right) monocytes among circulating mononuclear myeloid cells were tested for correlations with several pro-inflammatory cytokines and chemokines at week 0 (pre-ART) and at week 6 or at the time of IRIS after ART initiation in the entire study population (n = 48). Bars represent the strength of associations (r values), and P-values of statistically significant results are displayed.

These results support the idea that monocyte subsets are associated with the occurrence of TB-IRIS in HIV patients following ART initiation and, specifically, that circulating CD14^++^CD16^−^ cells are associated with IRIS pathogenesis. This was further supported by the fact that, in the entire Indian cohort (n = 48), the frequency of circulating CD14^++^CD16^−^ monocytes, but not other subsets, was strongly correlated with several key cytokines associated with inflammation and IRIS both at baseline (pre-ART) and after ART initiation (Figure 6).

**Figure 7:**
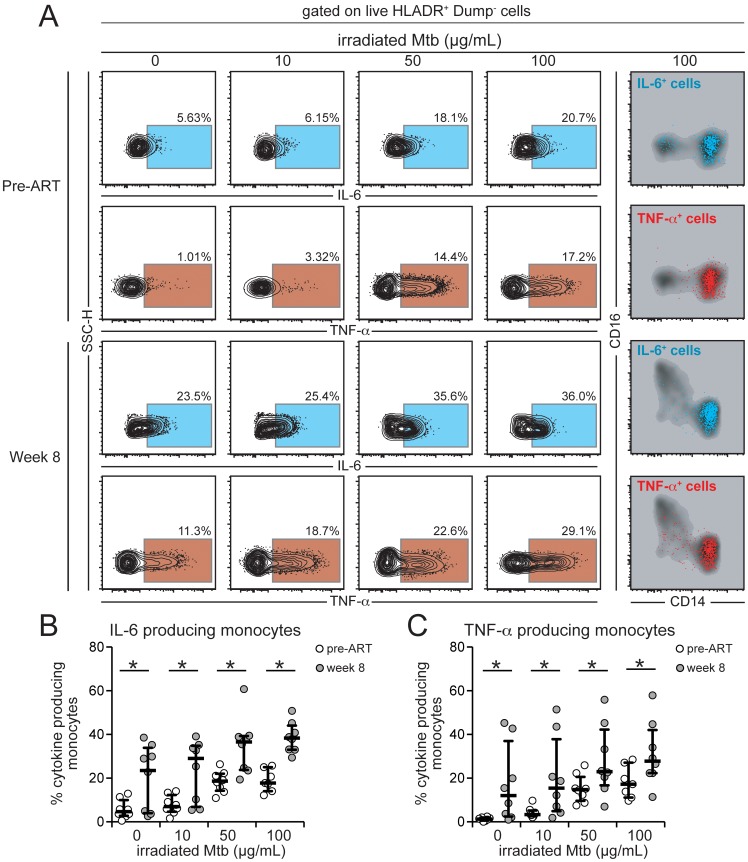
Intracellular production of IL-6 and TNF-α by monocytes from HIV+ patients is affected by HIV plasma viremia and mycobacterial antigen load. Paired PBMC samples from eight HIV-infected individuals prior to ART initiation and after 8 weeks of treatment when patients achieved virological suppression were incubated in vitro with different doses of irradiated *M. tuberculosis* (Mtb) for 6 h in the presence of brefeldin-A. Intracellular cytokine assays for detection of IL-6 and TNF-α in monocytes (HLA-DR^+^Dump^−^ cells) were performed by multicolor flow cytometry. Gates for cytokines were set up based on fluorescent minus one controls. (**A**) Representative FACS plots of percentage of monocytes expressing IL-6 or TNF-α (left panels) following stimulation with increasing doses of Mtb. Right panel shows overlays of CD14 vs. CD16 expression on cytokine-producing cells after stimulation with irradiated Mtb 100 µg/mL and reveals that most of the cytokine-producing cells in this *in vitro* system are CD14^++^CD16^−^ monocytes. (**B**) Percentage of monocytes from HIV+ patients with high or low viral loads stained positive for intracellular IL-6 after in vitro stimulation with different doses of irradiated Mtb. Data were analyzed using the Wilcoxon matched pairs test. (**C**) Percentage of monocytes from HIV+ patients with high or low viral loads stained positive for intracellular TNF-α after in vitro stimulation with different doses of irradiated Mtb. Data were analyzed using the Wilcoxon matched pairs test. * P<0.05.

Our results have demonstrated that CD14^++^CD16^−^ monocytes expand in the blood after ART initiation in patients who develop TB-IRIS (Figure 5 and Figure S3) and also correlate with the inflammatory milieu seen during the IRIS episode (Figure 6). We have also shown that the patients receiving ATT for shorter periods before ART initiation exhibited heightened Mtb sputum culture grades (suggestive of elevated mycobacterial burden) and displayed increased plasma concentration of several inflammatory cytokines associated with IRIS (Figure 2). These observations indicate that the mycobacterial load prior to immune reconstitution with ART may be critical to promote the inflammatory environment of IRIS. We hypothesized that elevated mycobacterial load leads to increased production of pro-inflammatory cytokines by monocytes in HIV infected patients responding to ART (after HIV viremia suppression). To test this hypothesis, we assessed the intracellular cytokine production by monocytes in North American HIV infected patients at pre-ART and week 8 post-ART initiation, who were not co-infected with Mtb (to control for differences of *in vivo* exposure to mycobacteria). We observed that the CD14^++^CD16^−^ monocyte subset represented the vast majority of IL-6 and TNF-α producing monocytes after Mtb stimulation (Figure 7A). Monocytes from HIV+ patients with suppressed viral load produced more intracellular IL-6 and TNF-α spontaneously and also upon stimulation with different doses of Mtb than those from individuals with HIV viremia (Figure 7B). These results argue that once HIV viral load is suppressed and CD4 cell numbers increase shortly after ART initiation, inflammatory monocytes can respond to Mtb with augmented production of IL-6 and TNF-α, cytokines tightly associated with IRIS.

**Figure 8:**
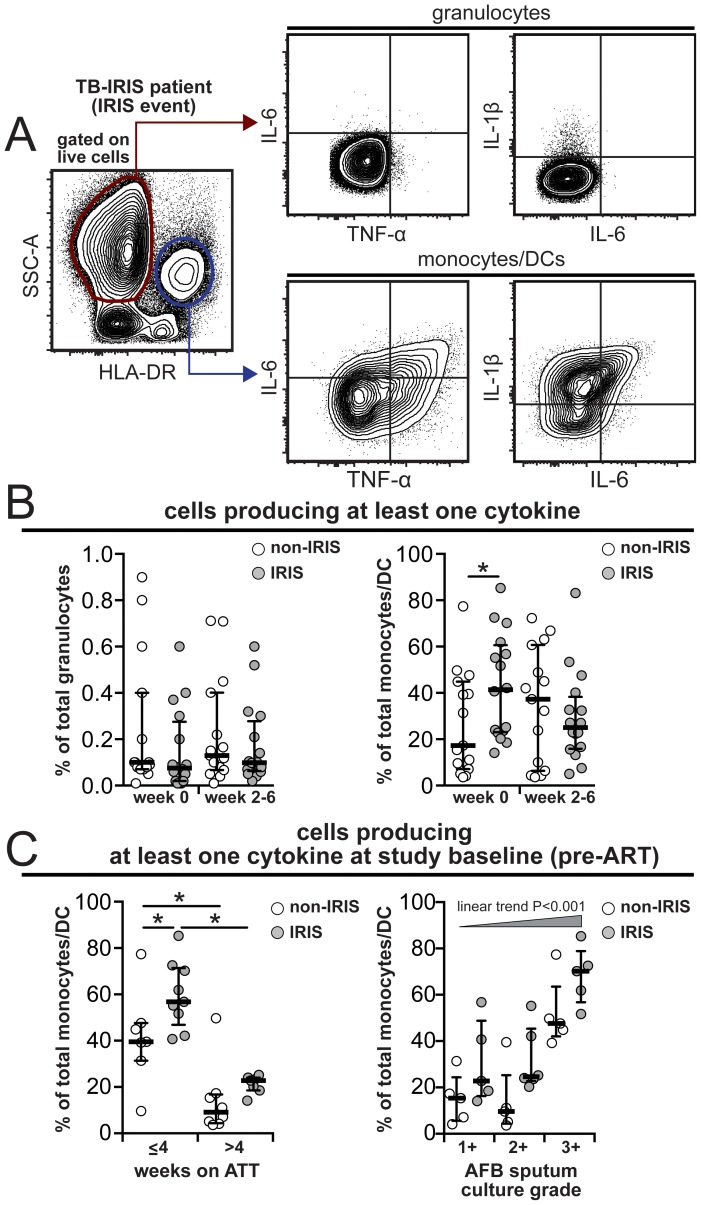
Increased frequency of monocytes spontaneously producing pro-inflammatory cytokines pre-ART in TB-IRIS patients. Whole blood intracellular cytokine assay was performed in 17 individuals from the IRIS group and 15 non-IRIS patients from the South Indian cohort. (**A**) Gating strategy to assess intracellular cytokine production by neutrophils and monocytes/DCs and (**B**) frequency of cells producing IL-1β, IL-6 and/or TNF-α after 6 hour *in vitro* culture in the presence of brefeldin-A (unstimulated). (**C**) Pre-ART frequency of monocytes producing IL-1β, IL-6 and/or TNF-α was compared between IRIS and non-IRIS patients stratified by time on ATT or *M. tuberculosis* loads in sputum cultures. Data were analyzed using Mann-Whitney or Wilcoxon matched-pairs test for paired analyses within each study group. * P<0.05, ** P<0.01, *** P<0.001.

Next, we directly tested if, aside from being strong predictors of IRIS, the CD14^++^CD16^−^ monocytes can produce key cytokines such as IL-1β, IL-6 and/or TNF-α in TB-IRIS patients. We performed intracellular cytokine assays in whole blood samples available from our South India Cohort (17 individuals who developed IRIS and 15 patients from the non-IRIS group). Monocytes, but not neutrophils, had high production of IL-1β, IL-6 and/or TNF-α when directly tested *ex vitro* (Figure 8A and Figure S4). Consistent with our observed associations, the cytokine producing monocytes seemed to be restricted within the CD14^++^CD16^−^ subset (Figure S4). Before ART initiation, IRIS patients exhibited an increased frequency of monocytes producing at least one of these pro-inflammatory cytokines than non-IRIS individuals (Figure 8B). At week 6 or IRIS event, there was no significant difference in the frequency of monocytes producing IL-1β, IL-6 and/or TNF-α (Figure 8B) although patients developing an IRIS event exhibited considerably increased frequency of total IL-1β and IL-6 producing cells, but not TNF-α, compared to non-IRIS patients. The increased frequency of monocytes producing pro-inflammatory cytokines prior to ART initiation was also strongly associated with a reduced time on ATT (≤4 weeks) and increased Mtb loads in sputum cultures (Figure 8C). These findings reinforce previous results associating higher pre-ART mycobacterial loads in IRIS patients [33] and strongly suggest that mycobacterial antigen load-driven monocyte activation contributes to systemic inflammation that predisposes to TB-IRIS.

## Discussion

Monocytes are a key component of the innate immune response and a critical link between innate and adaptive immunity. Despite the importance of monocytes in resistance to HIV infection and tuberculosis [34], [35], the role of monocyte subsets in TB-IRIS has not been systematically investigated. Results from transcriptional analyses recently suggested potential direct participation of monocytes in the pathogenesis of TB-IRIS [23], [24]. In the present study, soluble biomarkers of inflammation and monocyte activation were evaluated in patients with TB-HIV co-infection from India and South Africa, to examine the role of monocytes in pathogenesis of TB-IRIS. Our findings revealed distinct differences in monocyte subsets between individuals who developed paradoxical TB-IRIS compared to those who did not, which were strongly linked to the profound inflammatory milieu of IRIS.

Soluble markers derived from monocyte activation, such as sCD14, sCD163 and sTF have been extensively described in the context of HIV infection [36]–[40]. Plasma levels of sCD14 have been established as a strong indicator of monocyte activation [41] and as an independent predictor of death [38]. Here, we describe that pre-ART plasma levels of sCD14 increased at the time of IRIS, while decreasing in individuals not developing IRIS. In addition, patients in this study who developed IRIS already exhibited increased levels of CD163 pre-ART, which were sustained at high levels for up to 24 weeks after ART initiation. Levels of sCD163 positively correlated with the degree of cellular expression of CD163 on CD14^++^CD16^−^ monocytes, implying that this subset might be an important source of this marker in TB-IRIS. Our results showing an association between sCD163 and IRIS are not surprising, as this marker has been linked to a worse prognosis and increased HIV activity both prior to and after ART initiation [36] and appears to be an independent predictor of mortality in TB [37].

Activated monocytes in the circulation and tissue macrophages are also a major source of TF [42]. The shedding of TF by these cells triggers coagulation, which has been clearly associated with increased mortality and cardiovascular complications in HIV infection [39], [40]. More recently, it has been shown that Mtb induces TF expression in macrophages and Mtb signaling pathways that induce TF require cooperation of multiple receptors and co-factors including Toll-like receptors [43]. Our results demonstrate that levels of sTF in plasma are already elevated before ART initiation and increase even more in patients who develop IRIS upon immune reconstitution. Increased TF expression could be one of the factors that, together with sCD163, influence the onset of IRIS. More importantly, the analysis of plasma biomarkers of monocyte activation in this study favors the idea of increased monocyte activation prior to ART in patients who ultimately develop TB-IRIS and is corroborated by increased cytokine production by monocytes pre-ART in TB-IRIS patients.

There is now strong evidence that patients experiencing TB-IRIS exhibit increased systemic concentrations of multiple cytokines, chemokines and growth factors, a phenomenon known as cytokine storm or hypercytokinemia [20]. Our findings assessing multiple plasma biomarkers from patients originating from two different endemic countries for HIV-TB co-infection reinforce this idea. Interestingly, ATT itself seems to considerably affect the expression of several plasma biomarkers. At less than 4 weeks after TB treatment initiation, TB-HIV co-infected individuals exhibited increased concentrations of several plasma factors of innate immune origin, such as IL-1β, IL-6, IL-8 and CCL2. The levels of these and other innate soluble factors decreased with time on ATT, whereas markers of activation of adaptive immune responses significantly increased. We have previously shown for the same cohort of patients described here, that time from ATT to ART initiation was strongly associated with the occurrence of TB-IRIS, even after adjustment for several confounding factors, such as CD4^+^ T-cell counts and HIV viral load [30]. Because the majority of the IRIS patients were on ATT for a shorter period compared with non-IRIS controls, we speculate that the activated innate immune signature in these individuals may set the stage for paradoxical TB-IRIS upon ART initiation. More importantly, we describe that multiple soluble parameters measured in TB-IRIS patients during the event were not only increased in expression but also displayed strong positive correlations with each other, suggesting they belong to common activation pathways.

To our knowledge, this is the first study that formally describes monocyte subsets in an at-risk TB-HIV co-infected population and demonstrates associations between activation of these cellular subsets and TB-IRIS. Our results clearly show that the composition of monocyte subsets is significantly different between IRIS and non-IRIS patients, even before ART initiation. The striking finding that CD14^++^CD16^−^ and CD14^+^CD16^+^ monocytes are increased, whereas CD14^dim^CD16^+^ cells are dramatically decreased, in IRIS patients before ART initiation implies that the relative distribution of monocyte subsets may be an important predictor of IRIS. In addition, these results indicate that CD14^+^ monocytes or tissue myeloid cells derived from circulating monocytes might be the source of several of the inflammatory cytokines detected in plasma. The function of the different monocyte subsets in producing pro- or anti-inflammatory cytokines is controversial and has been variably reported in past studies, depending on the experimental design and inflammatory setting. Studies assessing sorted monocytes stimulated with different Toll-like receptor (TLR) ligands show that CD14^dim^CD16^+^ cells are more prone to producing TNF-α, whereas CD14^+^CD16^+^ cells are a major source of IL-10 [44], [45]. More recently, CD14^dim^CD16^+^ cells were shown to exhibit patrolling characteristics, with a weak ability for phagocytosis and a limited capacity to produce reactive oxygen species and cytokines when stimulated with bacterial TLR agonists but high amounts of TNF-α and IL-1β upon stimulation with viral ligands or nucleic acids [46]. These findings suggest that monocyte subsets cannot be categorized merely as pro- or anti-inflammatory and that their effector functions may depend on the activation pathway and immune microenvironment. Our study reveals that among the different types of monocytes, the CD14^++^CD16^−^ subset is the key monocyte population producing inflammatory cytokines and, upon ART initiation, has a clearly differentiated trajectory, expanding in TB-IRIS patients and proportionally decreasing in non-IRIS patients.

Macrophages derived from CD14^++^CD16^−^ monocytes have been shown to be more permissive to Mtb growth in vitro than those derived from the CD14^dim^CD16^+^ subset [47], due to increased c-maf expression. It is plausible that increased numbers of CD14^++^CD16^−^ monocytes of TB-HIV co-infected individuals with very low CD4^+^ T-cell counts (and, thus, with reduced capacity to provide co-stimulatory signals for antigen presenting cells) generate macrophages that are more permissive to Mtb infection and replication. When HIV-mediated immunosuppression is decreased upon ART initiation and antigen-specific CD4^+^ T-cells rapidly reconstitute, this may create a burst of T-cell help, with large numbers of infected macrophages now becoming fully activated simultaneously in individuals developing paradoxical TB-IRIS. This would result in a massive production of pro-inflammatory mediators, which contributes to IRIS immunopathology. We have proposed this hypothesis previously, based on observations from a murine model of mycobacterial IRIS in which similar changes in the same monocyte subsets are observed [17], [27], [28]. A pathogenic role of neutrophils was not supported by our data but was not studied exhaustively and should be further investigated.

In the current study, the percentage of circulating CD14^++^CD16^−^ monocytes, but not other subsets, was significantly correlated with several pro-inflammatory cytokines before and after ART initiation, reinforcing this hypothesis. In addition, we have also demonstrated here that CD14^++^CD16^−^ monocytes from HIV+ patients with suppressed viral replication are capable of producing larger amounts of IL-6 and TNF-α than at pre-ART upon stimulation with increasing doses of Mtb. Spontaneous direct *ex vivo* cytokine production was also higher pre-ART in TB-IRIS patients. These results may lead us to speculate that once HIV+ individuals with higher mycobacterial antigen loads suppress HIV replication, they become highly responsive to Mtb stimulation, which results in the production of pro-inflammatory cytokines that contribute to the activation of inflammatory pathways and the resultant immunopathology of TB-IRIS. The lack of differences in direct spontaneous cytokine production of monocytes from TB-IRIS patients at the IRIS time point may represent trafficking and maturation into tissue macrophages or myeloid dendritic cells, a hypothesis that should be further investigated. Understanding the key determinants from the innate immune system, and myeloid cells more specifically, that play a fundamental role in TB-IRIS can help develop better strategies for the prediction, clinical management and pharmacologic targeting of this important complication of ART.

## Materials and Methods

### Description of the patients from the South Indian cohort

The Indian TB-IRIS cohort study was nested within a randomized controlled trial (NCT 933790) at the National Institute for Research in Tuberculosis (NIRT), Chennai, enrolling HIV infected patients with newly diagnosed sputum culture-confirmed pulmonary TB, as previously reported [30]. The parent randomized controlled clinical trial (ongoing at the time of this report) is comparing daily vs. intermittent anti-TB therapeutic regimens in HIV infected patients with pulmonary TB [30]. Eligible participants were above 18 years of age, HIV infected, and initiating ART with pulmonary TB confirmed by sputum culture positive for rifampicin-sensitive *Mycobacterium tuberculosis* (Mtb). Clinical evaluations and blood collections were performed at baseline (pre-ART), at the time of IRIS event (between weeks 2–6 of ART) or after 6 weeks of ART in the non-IRIS group, and after 6 months of ART in both groups. Mycobacterial loads in sputum cultures were assessed as described elsewhere [30]. Patients were hospitalized for ART initiation and were discharged within two weeks. In this cohort, 48 individuals were enrolled and 26 (54%) developed IRIS during the study. IRIS events occurred at a median of 11 days (interquartile range, IQR: 7–16) after ART initiation. Patients who experienced IRIS were similar to those with uneventful follow-up (designated as controls who were also HIV-TB co-infected but did not develop IRIS) with regard to age, gender, body weight, and hepatic transaminase levels. In addition, CD4^+^ T-cell counts dramatically increased and plasma HIV viral load significantly decreased in all patients, and there was no clear difference in the magnitude of changes in these variables after the initiation of ART between patients who developed IRIS and those who did not [30]. The detailed clinical, laboratory, and microbiologic description of the study participants has been previously reported by our group [30].

### Description of the patients from the South African cohort

The South African cohort originated from a prospective observational study of hospitalized patients with HIV-associated TB conducted at Brooklyn Chest Hospital (Cape Town) between April 2009 and February 2011, as previously described [31], [32]. Inpatients at this hospital are transferred from other hospitals or referring TB clinics with an established diagnosis of severe TB and prescribed anti-TB treatment. In the parent study, HIV-TB patients were enrolled and followed for 12 weeks following ART initiation. Their median CD4 count was 55 cells/µL (IQR = 31–106) and median time from starting TB treatment to ART was 36 days (IQR = 27–57). A total of 112 patients were recruited, and 47 individuals (42%) developed paradoxical TB-IRIS, fulfilling INSHI case definitions [8] after a median of 12 days on ART (range 4–49). Collection of blood samples was performed at the study baseline (pre-ART) and at week 2 after ART initiation in both patients who developed IRIS and in those who did not during the follow up. Only ART-naïve patients were included in the immunological analyses (52 non-IRIS and 44 IRIS patients). A detailed description of the patients used in the immunological analyses is shown in Table S3.

### Description of the patients from the North American cohort

Paired cryopreserved PBMC samples from eight HIV-infected individuals non-infected with *Mycobacterium tuberculosis* prior to ART initiation (pre-ART) and after 8 weeks of ART initiation when patients achieved virological suppression were analyzed using intracellular cytokine assays. Patients had a median age of 32 years (IQR: 25–43 years), and 56% were female. Median pre-ART CD4^+^ T cell count was 32 cells/µL (IQR: 10–53 cells/µL), and median log_10_ HIV RNA level was 5.00 copies/mL (IQR: 4.46–5.27 copies/mL). At week 8 post-ART initiation (for 2 patients it was closer to week 12), CD4^+^ T-cell count was 82 cells/µL (IQR: 61–147 cells/µL), and median log_10_ HIV RNA level was 1.7 copies/mL (IQR: 1.7–1.8 copies/mL); these variables were significantly different from pre-ART values (P = 0.039 and P = 0.008, respectively).

### Ethics statement

All clinical investigations were conducted according to the principles expressed in the Declaration of Helsinki. Written informed consent was obtained from all participants before enrolling into the sub-studies. The Indian study was approved by the Scientific Advisory Committee and Institutional Ethics Committee of the National Institute for Research in Tuberculosis (Chennai) and is registered on Clinicaltrials.gov (NCT 933790). The South African study was approved by the University of Cape Town Faculty of Health Sciences Human Research Ethics Committee (HREC 049/2009). The North American study was approved by the NIAID Ethics committee, and the study is registered in Clinicaltrials.gov (NCT #00286767).

### Plasma biomarker measurements

Concentrations of IL-1β, IL-1Ra, IL-6, IL-8, IL-10, IL-12p40, IL-12p70, IL-15, CCL2, CCL3, CCL4, CCL5, CCL11, CXCL10, IFN-γ, TNF-α, TGF-β, platelet-derived growth factor (PDGF), vascular endothelial growth factor (VEGF) (Bio-Plex, Bio-Rad, Hercules, CA), C-reactive protein (CRP) (eBioscience, San Diego, CA), soluble (s) CD14, sCD163, soluble tissue factor (sTF) (R&D Systems, Minneapolis, MN) and intestinal fatty acid binding protein (I-FABP) (Hycult Biotech, The Netherlands) were assessed in cryopreserved plasma samples maintained at −80°C from patients in the Indian cohort. A smaller panel of biomarkers containing IL-6, IL-8, IL-10, IL-12p40, IFN-γ, TNF-α, CCL3, CCL4, CXCL10, sCD14, sCD163 and sTF was investigated in the South African cohort due to limitations in available plasma volume.

### Flow cytometry

The immunophenotyping of monocytes and dendritic cells (DC) was performed in whole blood collected in heparinized vacuum tubes. For *ex vivo* phenotyping, aliquots of 250 µL blood were stained with five panels of antibodies prepared in PBS 1% BSA for 1 h at room temperature (RT) to characterize the myeloid populations. The panels with antibody clones and fluorochromes as well as the gating strategies are listed in Figure S1. Antibodies were from eBioscience (San Diego, CA), Biolegend (San Diego, CA), BD Biosciences (San Jose, CA) and Life Technologies (Carlsbad, CA). Data from the Indian cohort were acquired on a BD FACS Canto II flow cytometer (BD Biosciences) and from the North American cohort were acquired on a BD LSR II flow cytometer (BD Biosciences). Three major monocyte subsets are considered for analysis based on CD14 and CD16 surface expression: classical/inflammatory (CD14^++^CD16^−^), intermediate (CD14^+^CD16+) and patrolling (CD14^dim^CD16^+^) monocytes. Data on monocyte subsets are displayed as percent of circulating mononuclear myeloid cells, defined in this study as HLA-DR^+^ Dump^−^ (CD2^−^CD3^−^CD19^−^CD20^−^CD56^−^) cells in the blood. All compensation and gating analyses were performed using FlowJo 9.5.3 (TreeStar, Ashland, OR).

### Intracellular cytokine assay

Frozen peripheral blood mononuclear cells (PBMC) were thawed and resuspended in RPMI-1640 media supplemented with 10% human AB serum, plated at 10^6^ cells/well in round bottom 96-well plates, and stimulated for 6 h with different concentrations of irradiated Mtb (0–100 µg/mL) in the presence of brefeldin-A at 37°C in 5% CO_2_. Cells were washed with PBS and incubated with Live/dead fixable blue dead cell stain (Life Technologies) for 20 min at RT, and then washed and stained with antibodies for surface markers. Cells were then fixed and permeabilized (Foxp3/Transcription Factor Staining Buffer Set, eBioscience) for 1 h at RT. After permeabilization, cells were stained for intracellular IL-6 and TNF-α (1 h at RT).

Spontaneous cytokine production by leukocytes in whole blood from TB-HIV co-infected patients was also assessed by incubating500 µL of fresh heparinized whole blood aliquots in polypropylene tubes at 37°C in 5% CO_2_, for 6 hours in the presence of Brefeldin A 5 µg/mL (BD Biosciences). Cells were fixed with 4% formaldehyde (Sigma Aldrich) and red cells lysed with BD FACS Lysing Solution (BD Biosciences). Cells were frozen in PBS 10% DMSO at −80°C and then at liquid nitrogen until all patients from the clinical study were recruited. All samples were thawed, re-suspended in FACS buffer and stained for surface markers. Cells were then permeabilized with eBioscience Permeabilization Buffer (eBiosciences) and stained for intracellular cytokines (IL-1β, IL-6 and TNF- α) and acquired in the flow cytometer.

### Network analysis

The inferential networks were generated from Spearman correlation matrices containing values of each biomarker measured in the plasma samples and blood cell counts (neutrophils and monocytes). The values were inputted in JMP 10.0 software (SAS, Cary, NC, USA). Each mediator is selected as a target, and the software performs a search within the other mediators for those that are correlated, with the target calculating a correlation matrix using Spearman rank tests. As a result, the features related to the selected target are linked. The links shown in the networks represent statistically significant Spearman rank correlations (P<0.05). In order to analyze the structure of the biomarker networks, the network density was calculated, which is, in the context of this study, the ratio of the number of edges inferred in the network over the total number of possible edges between all pairs of nodes [48]. The density measure is defined as follows: density = L/(N (N-1)/2), in which L is the number of observed edges (i.e., Spearman correlations with P<0.05) and N is the total number of the nodes in the network. The density is normalized, ranging between 0 (no edges in the network) and 1 (all possible edges presents). The number of statistically significant correlations was compared between the IRIS and non-IRIS groups at the different time points using the permutation test. The same test was used to compare the changes in the number of significant correlations in the networks from week 0 to week 6/IRIS event within the two clinical groups. Graphics for the network analysis were customized using the Ingenuity Systems Pathway Analysis software (Ingenuity Systems, Redwood City, CA, USA) and Adobe Illustrator (Adobe Systems Inc.).

### Data analysis

Median values with IQR or frequencies of variables were compared using the Mann-Whitney test (when two groups were compared) or the Kruskal-Wallis test with Dunn's multiple comparisons *ad hoc* analysis (when three groups were compared). Fisher's exact test or Chi-square tests were used to compare two or three groups, respectively, when data were displayed as percentages. Paired changes from before ART initiation to week 6 or the time of IRIS were compared using the Wilcoxon matched-pairs test. Using JMP 10.0 software, geometric mean values (log_10_) for each marker measured at week 0 and week 6 (Indian cohort) or week 2 (in South African cohort) were calculated for the entire study population. To assess the overall pattern of expression of these markers in each clinical group and time point, heat maps were built using variation from geometric mean values calculated for each candidate biomarker. A hierarchical cluster analysis using the Ward's method was employed to reveal the patterns of expression in plasma. The analyses were first performed in the Indian cohort and then repeated in the South African cohort for validation. The databank from South Africa contained a reduced number of biomarkers compared to India, and there was no pre-selection of markers based on the Indian cohort data. The relative risk (RR) of IRIS per standard deviation increase in the percentage of different monocyte subsets at week 0 (pre-ART) after log_10_ transformation was also calculated. RRs were adjusted for baseline age, gender, days to ART initiation, plasma HIV RNA levels and CD4^+^ T-cell count. Throughout the text, a statistically significant difference was defined as a P value<0.05. The statistical analyses were performed using GraphPad Prism 6.0 (GraphPad Software Inc., USA), STATA 9.0 (StataCorp, TX, USA), and JMP 10.0 software.

## Supporting Information

Data file S1 **Correlations from South Indian Cohort.**
(XLSX)

Data file S2 **Correlations from South African Cohort.**
(XLSX)

Figure S1 **Gating strategy used to assess monocytes.** (**A**) Antibody panels used to assess phenotype of monocytes and dendritic cells, as well as intracellular cytokine production by monocytes. (**B**) Gating strategy used to evaluate monocytes subsets in whole blood.
(TIF)

Figure S2 **Analysis of network densities of inflammatory biomarkers from TB-HIV co-infected patients from the Indian and South African cohorts.** Bars represent the network densities (calculated as described in Methods); symbols, and whiskers represent median values and interquartile ranges for plasma HIV viremia (left panels) or CD4^+^ T-cell counts from patients recruited in the Indian (**A**) or South African (**B**) cohorts. Plasma HIV-RNA and CD4^+^ T-cell counts were compared between patients at week 0 (pre-ART) and at week 2 after ART initiation using the Wilcoxon matched-pairs test (*** P<0.001). Differences between the network intensities were compared between week 0 and weeks 2–6/time of IRIS (# denotes P<0.05) or between IRIS and non-IRIS groups at each study time point († denotes P<0.05) using permutation tests.
(TIF)

Figure S3 **Expansion of circulating CD14^++^CD16^−^ monocytes is a hallmark of TB-IRIS in South India.** (**A**) Percentage of mononuclear myeloid cells (HLA-DR^+^CD2^−^CD3^−^CD19^−^CD20^−^CD56^−^) within total circulating leukocytes was compared at week 0 (pre-ART) and at weeks 6 or at the time of IRIS after ART initiation between TB-HIV co-infected patients that developed paradoxical TB-IRIS (n = 26) and those who did not (n = 22). Data were analyzed using the Mann-Whitney test or Wilcoxon matched-pairs test for analyses between and within study groups. (**B**) Pie charts (right panel) show median percentage of different circulating myeloid cell subsets at week 0 (pre-ART) and week 6 or at the time of IRIS after ART initiation. Bar graphs (right panel) show changes in percentage of the different myeloid cell subsets between week 6 and week 0 for IRIS vs. non-IRIS patients. Delta variations for each cellular subset were compared between IRIS and non-IRIS groups using the Mann-Whitney test. * P<0.05, ** P<0.01, *** P<0.001.
(TIF)

Figure S4 **Intracellular cytokine staining of circulating monocytes and neutrophils from TB-HIV infected patients.** (**A**) Refined gating strategy to identify neutrophils and monocytes used in the South Indian cohort. (**B**) Representative plots with median and IQR values of monocytes spontaneously producing IL-1β, IL-6 or TNF-α are shown for IRIS (n = 17) and non-IRIS patients (n = 15) at IRIS event or week 6, respectively. Frequencies of cytokine producing cells were compared between the groups using Mann-Whitney test.
(TIF)

Table S1 **Distribution of plasma biomarkers and cell counts in the Indian Cohort.**
(DOCX)

Table S2 **Distribution of plasma biomarkers and cell counts in the South African Cohort.**
(DOCX)

Table S3 **Baseline characteristics of the South African patients.**
(DOCX)
