# Supplementary figures and images for: Mycobacterial Antigen Driven Activation of CD14++CD16− Monocytes Is a Predictor of Tuberculosis-Associated Immune Reconstitution Inflammatory Syndrome
Source: PLoS Pathog. 2014 Oct 2;10(10):e1004433. doi: 10.1371/journal.ppat.1004433 (PMC4183698; doi:10.1371/journal.ppat.1004433)

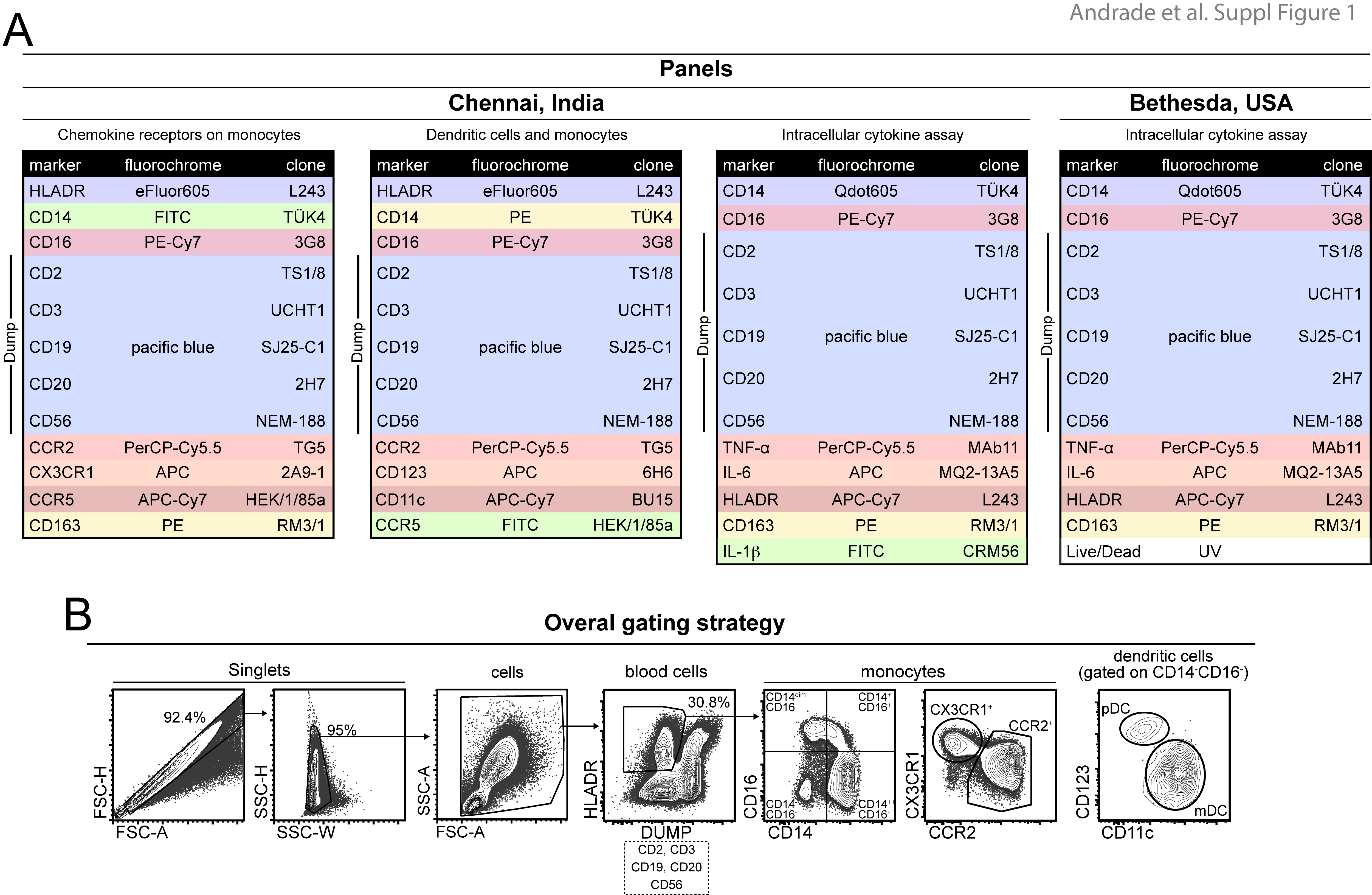

Supplement: Figure S1 — Gating strategy used to assess monocytes. (A) Antibody panels used to assess phenotype of monocytes and dendritic cells, as well as intracellular cytokine production by monocytes. (B) Gating strategy used to evaluate monocytes subsets in whole blood. (TIF) [file ppat.1004433.s003.tif]

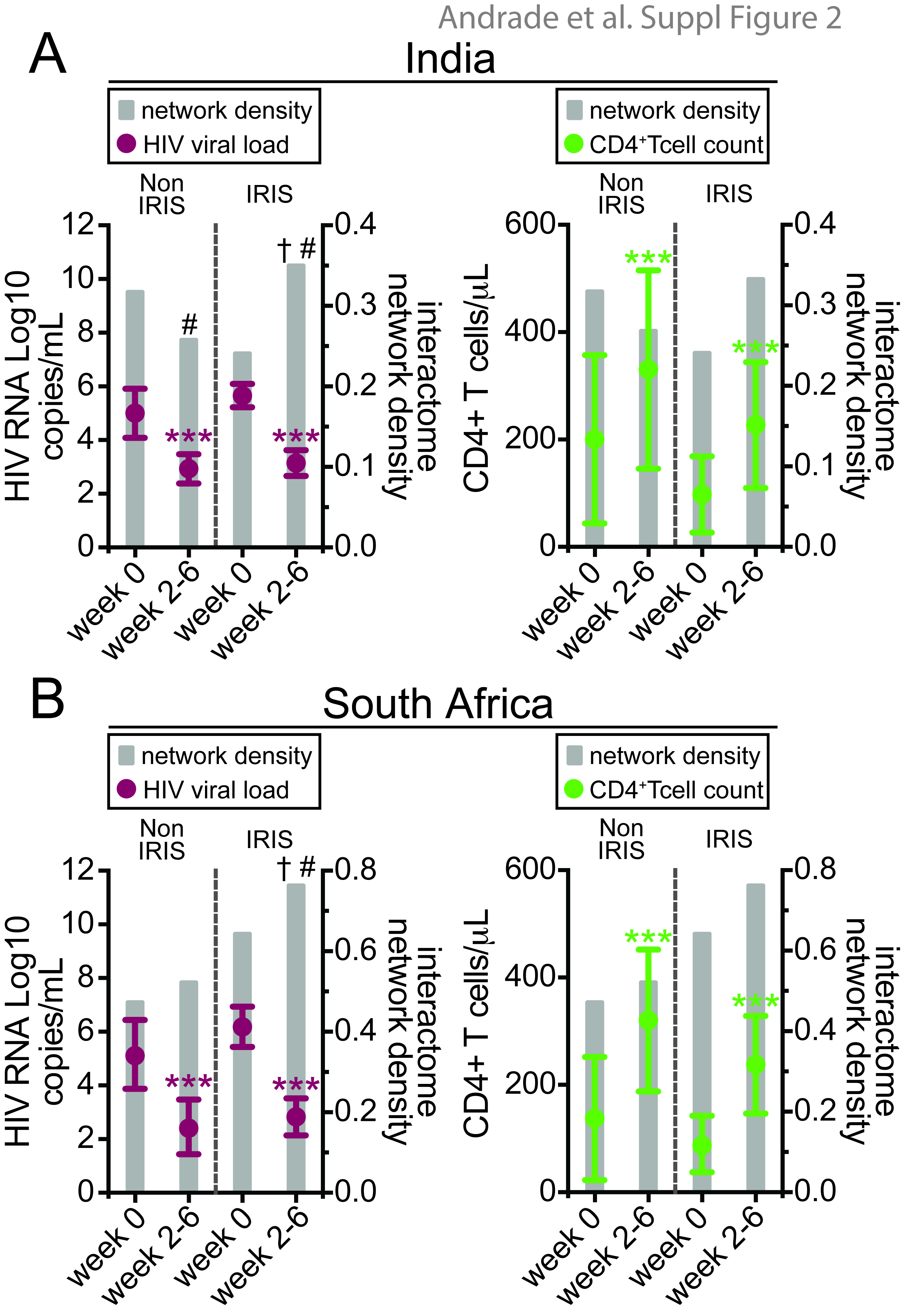

Supplement: Figure S2 — Analysis of network densities of inflammatory biomarkers from TB-HIV co-infected patients from the Indian and South African cohorts. Bars represent the network densities (calculated as described in Methods); symbols, and whiskers represent median values and interquartile ranges for plasma HIV viremia (left panels) or CD4+ T-cell counts from patients recruited in the Indian (A) or South African (B) cohorts. Plasma HIV-RNA and CD4+ T-cell counts were compared between patients at week 0 (pre-ART) and at week 2 after ART initiation using the Wilcoxon matched-pairs test (*** P<0.001). Differences between the network intensities were compared between week 0 and weeks 2–6/time of IRIS (# denotes P<0.05) or between IRIS and non-IRIS groups at each study time point († denotes P<0.05) using permutation tests. (TIF) [file ppat.1004433.s004.tif]

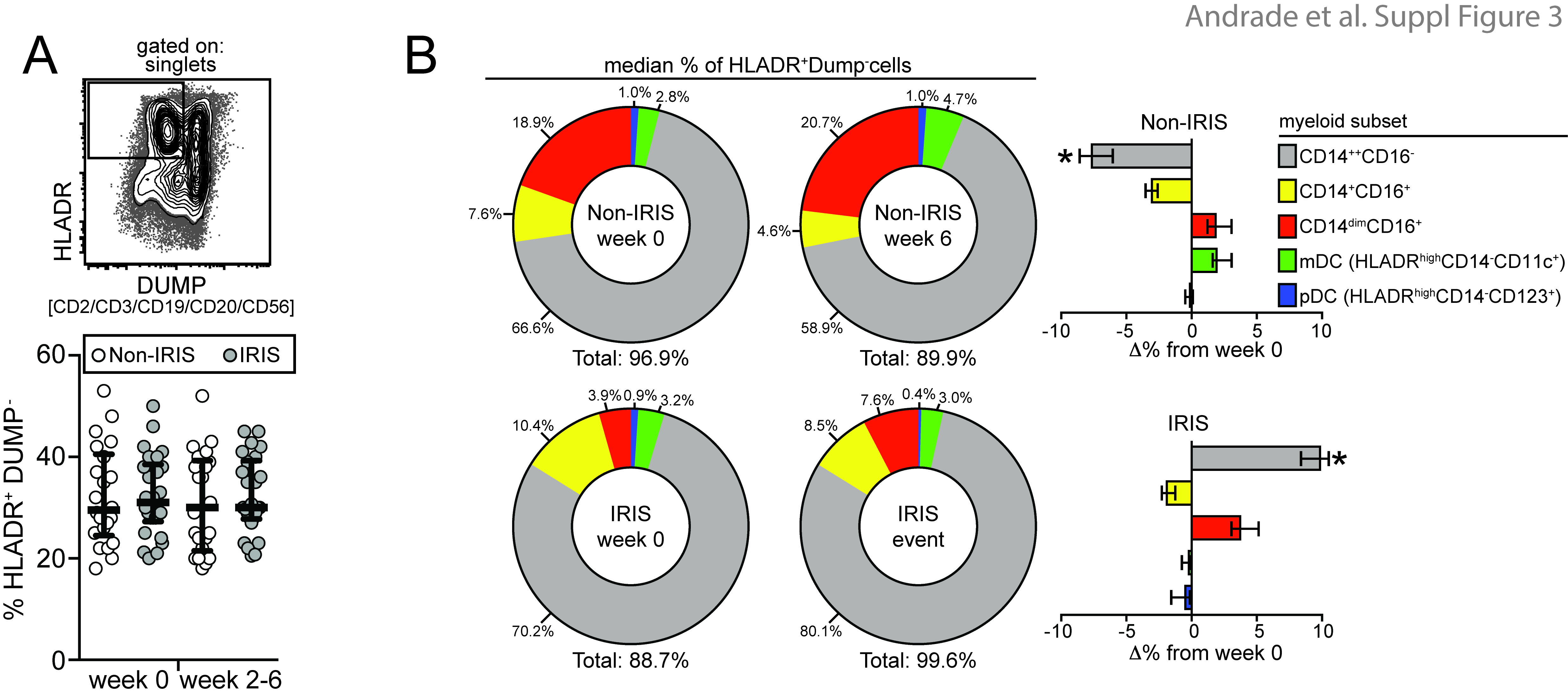

Supplement: Figure S3 — Expansion of circulating CD14++CD16− monocytes is a hallmark of TB-IRIS in South India. (A) Percentage of mononuclear myeloid cells (HLA-DR+CD2−CD3−CD19−CD20−CD56−) within total circulating leukocytes was compared at week 0 (pre-ART) and at weeks 6 or at the time of IRIS after ART initiation between TB-HIV co-infected patients that developed paradoxical TB-IRIS (n = 26) and those who did not (n = 22). Data were analyzed using the Mann-Whitney test or Wilcoxon matched-pairs test for analyses between and within study groups. (B) Pie charts (right panel) show median percentage of different circulating myeloid cell subsets at week 0 (pre-ART) and week 6 or at the time of IRIS after ART initiation. Bar graphs (right panel) show changes in percentage of the different myeloid cell subsets between week 6 and week 0 for IRIS vs. non-IRIS patients. Delta variations for each cellular subset were compared between IRIS and non-IRIS groups using the Mann-Whitney test. * P<0.05, ** P<0.01, *** P<0.001. (TIF) [file ppat.1004433.s005.tif]

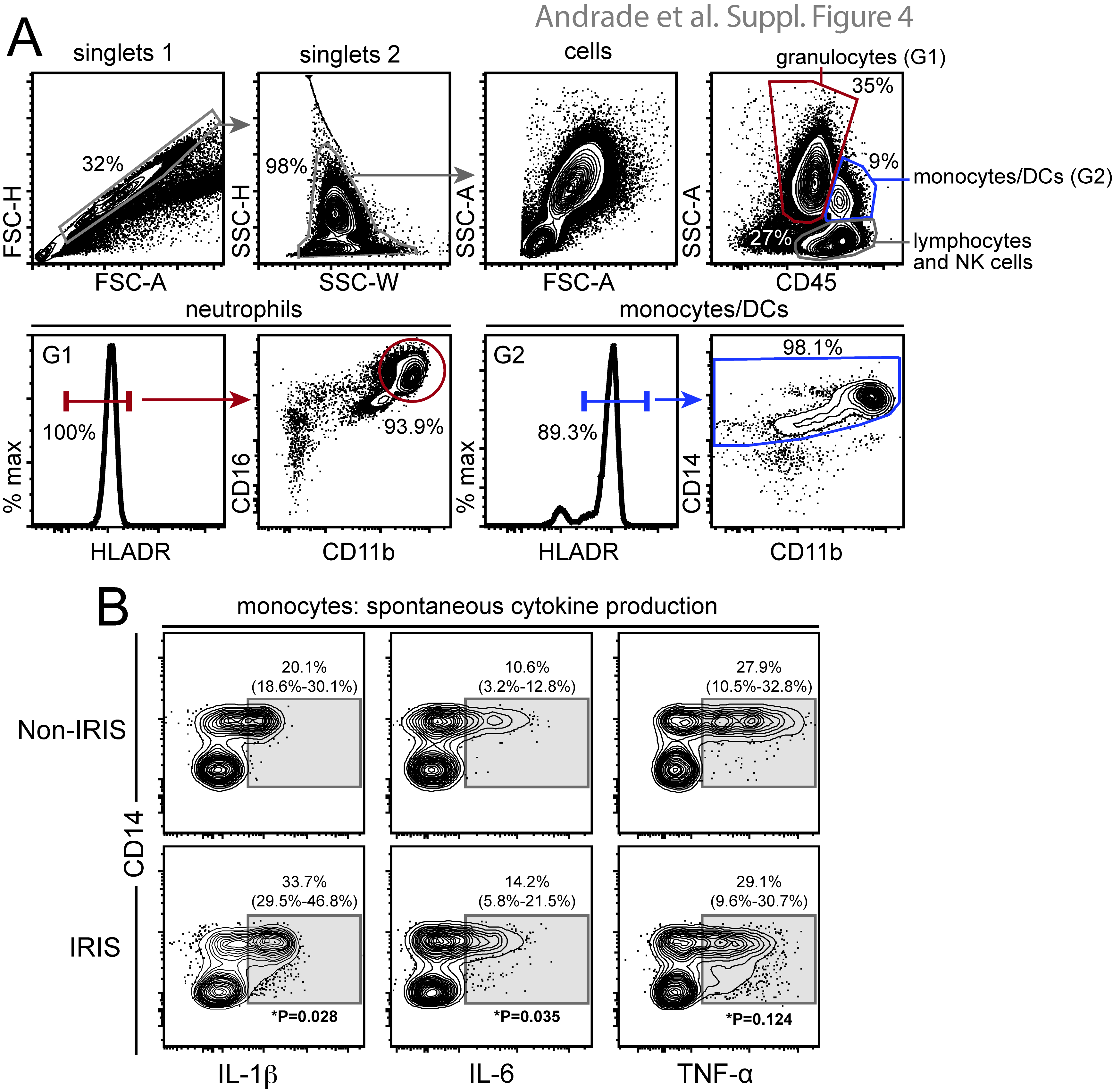

Supplement: Figure S4 — Intracellular cytokine staining of circulating monocytes and neutrophils from TB-HIV infected patients. (A) Refined gating strategy to identify neutrophils and monocytes used in the South Indian cohort. (B) Representative plots with median and IQR values of monocytes spontaneously producing IL-1β, IL-6 or TNF-α are shown for IRIS (n = 17) and non-IRIS patients (n = 15) at IRIS event or week 6, respectively. Frequencies of cytokine producing cells were compared between the groups using Mann-Whitney test. (TIF) [file ppat.1004433.s006.tif]
