# Supplementary material for: Mycobacterial Antigen Driven Activation of CD14++CD16− Monocytes Is a Predictor of Tuberculosis-Associated Immune Reconstitution Inflammatory Syndrome
Source: PLoS Pathog. 2014 Oct 2;10(10):e1004433. doi: 10.1371/journal.ppat.1004433 (PMC4183698; doi:10.1371/journal.ppat.1004433)
Supplement: Table S1 — Distribution of plasma biomarkers and cell counts in the Indian Cohort. (DOCX) [file ppat.1004433.s007.docx]

**Table S1. Distribution of plasma biomarkers and cell counts in the Indian Cohort**

| **Parameter** | | **Unit** | | **Non-IRIS**  **week 0** | | **IRIS**  **week 0** | **P value** | **Non-IRIS**  **week 6** | | **IRIS**  **event** | | **P-value** | |  |
| --- | --- | --- | --- | --- | --- | --- | --- | --- | --- | --- | --- | --- | --- | --- |
| ***Chemokines*** | CCL2 | | pg/mL | | 31.9  (14.0-59.2) | 39.9  (18.074.9) | 0.620 | | 23.0  (12.3-34.2) | | 29.7  (10.3-91.9) | | 0.702 | |
|  | CCL3 | | pg/mL | | 8.7  (3.9-19.1) | 13.2  (7.3-29.5) | 0.162 | | 8.7  (4.7-10.7) | | 18.6  (6.5-108.0) | | **0.026** | |
|  | CCL4 | | pg/mL | | 78.8  (51.9-157.9) | 99.7  (81.9-180.1) | 0.232 | | 89.0  (60.2-123.5) | | 172.4  (78.7-541.1) | | 0.069 | |
|  | CCL5 | | ng/mL | | 1.86  (1.48-2.06) | 1.7  (0.7-2.1) | 0.650 | | 1.0  (0.66-2.0) | | 1.2  0.5-2.1) | | 0.740 | |
|  | CCL11 | | pg/mL | | 79.8  (47.6-120.9) | 117.7  (87.7-153.9) | 0.051 | | 93.6  (42.1-123.1) | | 90.5  (63.1-183.4) | | 0.520 | |
|  | CXCL10 | | ng/mL | | 20.4  (10.1-25.1) | 12.3  (8.5-19.3) | 0.185 | | 8.3  (5.9-13.1) | | 14.6  (4.7-24.4) | | 0.155 | |
| ***Cytokines*** | IL-1β | | pg/mL | | 1.3  (0.8-2.7) | 2.4  (1.0-8.7) | 0.142 | | 0.8  (0.6-2.2) | | 13.2  (8.3-21.3) | | **0.001** | |
|  | IL-1Ra | | pg/mL | | 74.4  (42.5-101.7) | 68.7  (54.6-146.6) | 0.427 | | 38.9  (27.0-61.9) | | 75.5  (43.9-197.2) | | **0.035** | |
|  | IL-6 | | pg/mL | | 14.6  (9.8-20.4) | 39.8  (22.5-77.5) | **<0.001** | | 10.4  (4.6-21.0) | | 50.9  (25.0-198.9) | | **<0.001** | |
|  | IL-8 | | pg/mL | | 94.8  (29.1-283.6) | 169.1  (52.7-406.7) | 0.371 | | 53.3  (33.2-115.9) | | 155.5  (60.1-626.3) | | 0.072 | |
|  | IL-10 | | pg/mL | | 9.6  (5.9-10.9) | 11.0  (6.6-14.7) | 0.408 | | 8.6  (4.9-10.6) | | 14.7  (6.1-17.8) | | 0.238 | |
|  | IL-12p70 | | pg/mL | | 39.8  (22.5-54.7) | 33.9  (23.6-51.9) | 0.612 | | 36.0  (27.9-41.0) | | 29.2  (20.3-43.5) | | 0.363 | |
|  | IL-15 | | pg/mL | | 3.3  (1.9-8.5) | 2.5  (1.0-6.4) | 0.312 | | 5.8  (1.1-6.2) | | 5.6  (2.3-10.4) | | 0.416 | |
|  | IFN-γ | | pg/mL | | 104.0  (80.0-121.2) | 112.8  (86.0-155.5) | 0.274 | | 68.0  (59.5-93.9) | | 104.5  (79.3-160.3) | | **0.013** | |
|  | TGF-β | | pg/mL | | 607.1  (572.6-834.5) | 897.5  (613.9-1239) | **0.040** | | 602.5  (470.6-748.7) | | 823.5  (679.6-975.5) | | **0.026** | |
|  | TNF-α | | pg/mL | | 4.5  (3.9-13.7) | 11.8  (4.3-21.3) | **0.035** | | 2.0  (0.7-7.6) | | 26.5  (4.0-45.6) | | **0.015** | |
| ***Other biomarkers*** | CRP | | mg/L | | 47.5  (28.1-101.6) | 115.5  (79.9-194.1) | **<0.001** | | 19.0  (6.7-37.5) | | 108.0  (42-229) | | **<0.001** | |
|  | I-FABP | | pg/mL | | 221.1  (116.7-312) | 297.3  (174.2-390.7) | 0.179 | | 174.1  (97.4-226.9) | | 224.1  (176.9-309.2) | | 0.268 | |
|  | PDGF | | ng/mL | | 4.5  (1.4-5.5) | 2.9  (0.9-4.2) | 0.209 | | 2.5  (0.6-4.4) | | 1.9  (0.3-4.4) | | 0.797 | |
|  | VEGF | | pg/mL | | 159.3  (107.7-354.7) | 254.2  (88.7-597.1) | 0.473 | | 134.7  (59.0-251.0) | | 185.5  (65.9-593.1) | | 0.460 | |
|  | sCD14 | | μg/mL | | 5.5  (4.4-6.7) | 3.8  (3.3-4.4) | **<0.001** | | 2.6  (2.0-5.7) | | 5.7  (4.0-8.5) | | **<0.001** | |
|  | sCD163 | | ng/mL | | 3257  (3022-3524) | 3638  (3411-3880) | **0.001** | | 3058  (2848-3256) | | 3661  (3239-3952) | | **<0.001** | |
|  | sTF | | pg/mL | | 13.9  (10.2-28.2) | 32.5  (19.3-52.1) | **0.031** | | 21.9  (12.1-30.7) | | 66.6  (54-135.6) | | **<0.001** | |
| ***Cell counts*** | Monocytes | | cells/μL | | 467.0  (383.3-531.9) | 578.2  (386.1-961.7) | 0.109 | | 472.7  (397.2-623.3) | | 602.1  (401.7-781.1) | | 0.379 | |
|  | Neutrophils | | cells/μL | | 4660  (2428-6660) | 3087  (2517-4601) | 0.209 | | 2654  (2067-3514) | | 5767  (3500-7578) | | **0.002** | |

Median values with interquartile ranges are shown. Data were analyzed using the Mann-Whitney test and statistically significant P-values are shown in bold font.
