# Supplementary material for: Mycobacterial Antigen Driven Activation of CD14++CD16− Monocytes Is a Predictor of Tuberculosis-Associated Immune Reconstitution Inflammatory Syndrome
Source: PLoS Pathog. 2014 Oct 2;10(10):e1004433. doi: 10.1371/journal.ppat.1004433 (PMC4183698; doi:10.1371/journal.ppat.1004433)
Supplement: Table S2 — Distribution of plasma biomarkers and cell counts in the South African Cohort. (DOCX) [file ppat.1004433.s008.docx]

**Table S2. Distribution of plasma biomarkers and cell counts in the South African Cohort**

| **Parameter** | | | **Unit** | | **Non-IRIS**  **week 0** | | **IRIS**  **week 0** | | **P value** | | **Non-IRIS**  **week 2** | | **IRIS**  **week 2** | | **P-value** | |
| --- | --- | --- | --- | --- | --- | --- | --- | --- | --- | --- | --- | --- | --- | --- | --- | --- |
| ***Chemokines*** | CCL3 | pg/mL | | 1.6  (0-24.6) | | 16.3  (0-33.8) | | 0.268 | | 4.7  (0-32.9) | | 24.7  (0-37.9) | | 0.223 | |  |
|  | CCL4 | pg/mL | | 30.5  (20.4-53.4) | | 32.8  (26.4-55.0) | | 0.314 | | 43.3  (30.5-68.2) | | 52.6  (32.6-72.3) | | 0.512 | |  |
|  | CXCL10 | ng/mL | | 5.1  (2.7-10.5) | | 5.5  (3.4-9.3) | | 0.454 | | 4.9  (2.8-13.7) | | 10.2  (3.3-18.5) | | 0.072 | |  |
| ***Cytokines*** | IL-6 | pg/mL | | 3.6  (0-10.1) | | 4.54  (0-12.6) | | 0.902 | | 4.7  (0-10.9) | | 19.3  (0-31.5) | | **0.011** | |  |
|  | IL-8 | pg/mL | | 18.1  (10.4-35.0) | | 22.6  (14.8-38.6) | | 0.201 | | 17.0  (9.6-34.0) | | 41.1  (24.3-82.4) | | **<0.001** | |  |
|  | IL-10 | pg/mL | | 12.2  (4.2-24.4) | | 20.8  (10.1-33.9) | | **0.022** | | 12.9  (0-24.2) | | 16.1  (12.4-26.1) | | 0.154 | |  |
|  | IL-12p40 | pg/mL | | 0  (0-0) | | 13.2  (0-36.5) | | 0.287 | | 8.2  (0-27.3) | | 17.9  (0-43.6) | | 0.118 | |  |
|  | IFN-γ | pg/mL | | 8.8  (3.6-23.8) | | 24.3  (5.2-50.4) | | **0.021** | | 8.8  (2.3-26.7) | | 43.4  (10.1-62.2) | | **<0.001** | |  |
|  | TNF-α | pg/mL | | 31.2  (23.7-52.3) | | 40.9  (29.1-59.0) | | 0.186 | | 38.1  (23.7-68.5) | | 78.1  (39.3-108.5) | | **0.002** | |  |
| ***Other biomarkers*** | CRP | mg/L | | 24.6  (6.8-51.2) | | 10.6  (3.8-23.1) | | **0.012** | | 28.1  (13.9-46.7) | | 76.6  (17.7-175.5) | | **0.041** | |  |
|  | sCD14 | μg/mL | | 4.1  (3.1-4.6) | | 3.7  (3.0-6.2) | | 0.502 | | 3.2  (2.8-4.0) | | 4.4  (3.2-6.2) | | **0.003** | |  |
|  | sCD163 | ng/mL | | 1611  (1473-1692) | | 1946  (1543-2481) | | **0.005** | | 1630  (1533-1739) | | 2005  (1676-2499) | | **0.003** | |  |
|  | sTF | pg/mL | | 13.9  (3.6-28.9) | | 14.5  (7.7-24.7) | | 0.124 | | 15.2  (5.0-31.3) | | 20.0  (8.7-32.3) | | 0.085 | |  |
| ***Cell counts*** | Monocytes | cells/μL | | 330.0  (210.0-490.0) | | 290.0  (150.0-430.0) | | 0.718 | | 280.0  (212.5-465.0) | | 370.0  (230.0-530.0) | | 0.401 | |  |
|  | Neutrophils | cells/μL | | 2690  (1870-4750) | | 3270  (2150-5130) | | 0.283 | | 3375  (2065-6595) | | 5160  (3590-6460) | | **0.037** | |  |

Median values with interquartile ranges are shown. Data were analyzed using the Mann-Whitney test and statistically significant P values are shown in bold font.
