# Supplementary material for: Mycobacterial Antigen Driven Activation of CD14++CD16− Monocytes Is a Predictor of Tuberculosis-Associated Immune Reconstitution Inflammatory Syndrome
Source: PLoS Pathog. 2014 Oct 2;10(10):e1004433. doi: 10.1371/journal.ppat.1004433 (PMC4183698; doi:10.1371/journal.ppat.1004433)
Supplement: Table S3 — Baseline characteristics of the South African patients. (DOCX) [file ppat.1004433.s009.docx]

**Table S3. Baseline characteristics of the South African patients.**

| **Characteristic** | **IRIS**  **(n=47)** | **Non-IRIS**  **(n=52)** | **P-Value** |
| --- | --- | --- | --- |
| Male gender **–** no. **(%)** | 19 (40.4%) | 22 (42.3%) | > 0.999 |
| Age, median years (IQR) | 33.7 (28.6-41.6) | 32.3 (26.3-40.3) | 0.359 |
| Time to ART, median days (IQR) | 36.0 (27.0-57.0) | 35.0 (26.0-56.8) | 0.945 |
| Hemoglobin, median g/dL (IQR) | 9.0 (8.2-10.4) | 9.1 (8.0-10.1) | 0.918 |
| CD4^+^ T cells/µL, median (IQR) | 9.2 (8.2-10.1) | 44.0 (31.0-108.0) | **<0.001** |
| HIV RNA, median log_10_ copies/mL plasma (IQR) | 5.83 (5.32-6.15) | 5.69 (5.16-6.24) | 0.867 |
| Presence of extrapulmonary TB site – no. (%) | 40 (85.1%) | 44 (84.6%) | > 0.999 |

Median values with interquartile ranges or frequencies (percentage) are shown. Data were analyzed using the Mann-Whitney test, except for frequency of male and presence of extrapulmonary TB site, which were assessed using Fisher’s exact test. Statistically significant P values are shown in bold font.
